# Current opinion on large-scale prospective myomectomy databases toward evidence-based preconception and antenatal counselling utilising a standardised myomectomy operation note

**DOI:** 10.52054/FVVO.16.4.006

**Published:** 2024-03-28

**Authors:** S.M. Strong, A.A. McDougall, A.M. Abdelmohsen, A Maku, A Dehnel, R Mallick, F Odejinmi

**Affiliations:** Whipps Cross University Hospital, Whipps Cross Road, London, E11 1NR, UK; Homerton University Hospital, Homerton Row, London, E9 6SR, UK; William Harvey Hospital, Kennington Road, Ashford, Kent TN24 0LZ, UK; Medway Maritime Hospital, Windmill Road, Gillingham, Kent ME7 5NY, UK; Maidstone Tunbridge Wells NHS Trust, Hermitage Lane, Maidstone, Kent, ME16 9QQ, UK; Brighton and Sussex University Hospitals NHS Trust, Kemptown Brighton, BN2 1ES, UK

**Keywords:** Leiomyoma, Minimal Access Surgery, Uterine Rupture, Antenatal, Fibroids

## Abstract

**Background:**

No large-scale databases exist of pregnancy outcomes and rate of uterine rupture for women after myomectomy, resulting in inconsistent antenatal counselling and decision-making regarding mode and timing of delivery. Standardising information collected at myomectomy may facilitate data collection, informing prenatal/ antenatal counselling.

**Objectives:**

To determine clinician opinions regarding standardisation of myomectomy operation notes to allow comprehensive data input into a prospective database of pregnancy outcomes, toward an evidence-based approach to decision making regarding timing and mode of delivery in subsequent pregnancies.

**Materials and Methods:**

A google forms survey was emailed to all consultant (attending-level) obstetricians and gynaecologists across 25 hospitals in London, Kent, Surrey, and Sussex (UK) between March and May 2022. To enhance response rates, two further email reminders were sent alongside in-person reminders from selected local unit representatives.

**Main outcome measures:**

Senior clinician opinion for characteristics necessary to collect at time of surgery to develop a widescale database of post myomectomy pregnancy outcomes.

**Results:**

209/475 (44%) responses received; 95% (198/209) agreed with standardising operation notes. Criteria selected for inclusion included cavity breach (98%, 194/198), location (98%, 194/198), number of fibroids removed (93%, 185/198) and number of uterine incisions (96%, 190/198).

**Conclusions:**

Gynaecologists support standardising myomectomy operation notes to inform the development of prospective large-scale databases of pregnancy outcomes after myomectomy.

**What is new?:**

Acquisition of clinician opinions on the development and content of a standardised myomectomy operation note to aid the development of a pregnancy-outcome database for women after myomectomy.

## Introduction

There remains a paucity of data regarding pregnancy outcomes for women with previous myomectomy such as suggested mode of delivery, timing of delivery and rate of uterine rupture (Landon and Lynch, 2011). As women increasingly delay conception until later in life (Donnez, 2021), uterine preserving techniques for management of fibroids are often a priority for patients (Solberg et al., 2009). Grainger et al. (2023) recently reported a preference for vaginal birth amongst the majority of patients surveyed, who conceived post myomectomy, and highlighted the need for a shared decision-making model. However, patients continue to receive conflicting antenatal counselling resulting from a lack of evidence in this area (McDougall et al., 2023), making the choice of mode of delivery a challenging decision (Delli Carpini et al., 2023). The lack of data likely stems from ethics of randomisation of women in clinical trials like the parachute dilemma (Smith and Pell, 2003); the heterogeneity of presentation and demographics of women with fibroids, as well as the nature, location, and symptoms experienced as a result of the fibroids themselves.

For clinicians to provide evidence-based counselling, pregnancy outcome registries in combination with surgical characteristics and patient demographics are essential to allow for a comparison of outcomes (Stuart et al., 2015). We propose that the first step in achieving this is by designing a standardised operation note to capture surgical and patient data to input into a widescale database.

We performed a comprehensive survey of clinicians’ opinions regarding which information they felt should be documented in a proposed standardised operation note as a data collection tool to help determine the association between patient and operative characteristics at time of myomectomy and risk of uterine rupture and other obstetric complications in future pregnancies, to shape prenatal and antenatal counselling.

## Materials and Methods

We designed and distributed an electronic Google forms questionnaire based on guidance from Burns et al. (2008) with a key objective identified and purposive sampling techniques. Item generation was initiated by the collaborating authors working at separate units (FO/RM) who regularly perform high volumes of laparoscopic and open myomectomies as part of their practice following a literature review to identify potential associations with uterine rupture after myomectomy (Odejinmi et al., 2020).

The survey was designed to best capture expert consensus on what factors of a previous open or laparoscopic myomectomy should be included in an operation note that would influence antenatal counselling regarding mode of delivery in a subsequent pregnancy (i.e., which factors when combined with patient characteristics may predict the future risk of obstetric complications, specifically uterine rupture risk). A free-text comments section was included to allow for further expert opinion.

To develop the questionnaire, an initial list of suggested questions by FO/RM were generated. One senior obstetrics and gynaecology doctor was selected as a unit representative from each of the 25 sites, to enhance local return rates of the survey. Each unit representative was invited to participate in focus group meetings to share ideas, facilitate question item reduction, confirm question clarity, further assist with grouping questions by common stems for ease of completion and to pilot-test the questionnaire. All authors contributed to and approved the final version of the survey before distribution.

Once revised and agreed, the survey was sent to all consultant (attending level) obstetricians and gynaecologists across 25 hospitals in London, Kent, Surrey, and Sussex (United Kingdom) via email with a link to complete the survey by the individual unit trainee representative at each site. The survey remained open for 8 weeks (07/03/2022 to 07/05/2022). To enhance response rates, two further email reminders were sent alongside in- person reminders from the selected local unit representatives.

The electronic Google forms survey consisted of 22 questions assessing physician demographics, factors influencing their decision making for supporting or discouraging TOLAM, which surgical and patient characteristics/demographics should be included in a standardised operation note to assess the association of myomectomy with the risk of obstetric complications/uterine rupture, and opinion on standardising myomectomy operation notes and enrolling patient data into prospective databases.

A threshold of 60% (majority vote) was chosen to select which components should be included in the design of a standardised operation note.

Data were collected using Google forms and collected on an Excel spreadsheet. These were then reviewed by authors FO/RM/SS/AM to design an example standardised operation note highlighting those factors deemed essential by majority vote (>60%) to include (highlighted with an asterisk) with a view to establishing a myomectomy database to allow for correlation with pregnancy outcomes (Appendix 1). The example operation note also included standardised information routinely recorded in our local units’ operation notes, which are important to record as a surgical record, though do not serve a purpose to shaping a myomectomy database.

With regards to ethical approval, the HRA decision tool was used, and NHS REC review was deemed not to be required.

## Results

**Figure 1:**
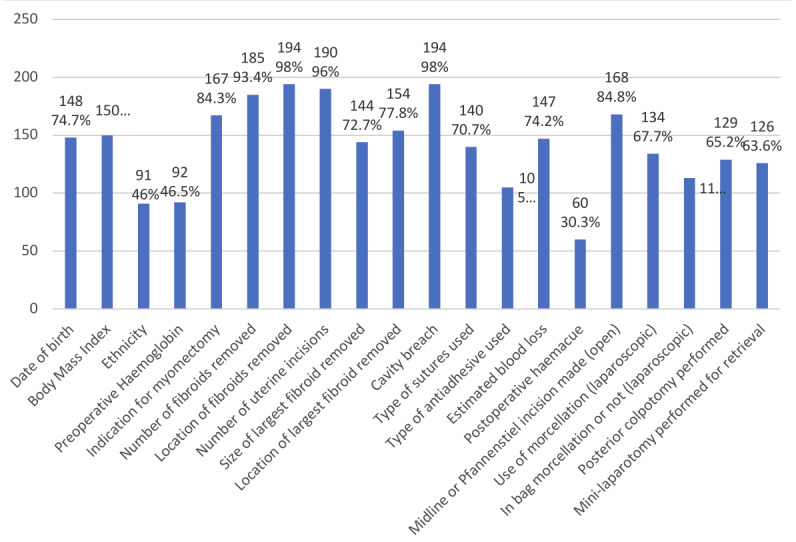
Components chosen by survey respondents to be included in a standardised operation note for myomectomy (n=198). Red dashed line represents the 60% threshold inclusion rate chosen by clinicians.

Survey responses were received between 7/3/2022 - 7/5/2022. The majority (77%, 161/209) worked both as an obstetrician and gynaecologist, 10% (21/109) were pure gynaecologists and 13% (27/209) pure obstetricians. Almost all clinicians (95%, 198/209) supported the implementation of a standardised operation note for myomectomy. Aspects that were most popularly favoured to include in a standard operation note were breach of the cavity (98%, 194/198), location of fibroids removed (98%, 194/198), number of uterine incisions made (96%, 190/198) and number of fibroids removed (93%, 185/198). Responders felt that certain information was not relevant to include in a standardised operation note (less than 50% of practitioners favouring their inclusion) such as patient’s ethnicity (46% 91/198) and documentation of preoperative haemoglobin level (46.5% 92/198). Figure 1 shows the components chosen by survey respondents to be included in a standardised operation note.

173/209 (82.8%) of survey respondents answered that they would support enrolling patients into a prospective database to help determine which factors at time of myomectomy influence risks in future pregnancies.

## Discussion

Prospective data of pregnancy outcomes after myomectomy needs to be collected to enable the provision of evidence-based counselling to women for future pregnancies. We have shown that there is support for the development of a standardised myomectomy operation note to inform the development of a prospective, multicentre database of pregnancy outcomes after myomectomy. Collection of uniform information in this way can facilitate the collection of prospective and comprehensive high-quality data and associate these with data of pregnancy outcomes. In this way prognosis can be better predicted, especially regarding factors related to the risk of uterine rupture.

To date, no single operative factor for uterine rupture has been identified (Parker et al., 2010) following laparoscopic myomectomy. Claeys et al. (2014) were also unable to quantify the risk of uterine rupture based on the method of myomectomy with the data available at that time. Thus, the need to collect standardised operation data is clear. Based on the results of our survey and expert author opinion following literature review, factors considered key to assessing who is more likely to have a uterine rupture are 1) whether the uterine cavity was breached, 2) number, location and size of uterine incisions made, 3) number and size of fibroids removed, 4) number of layers of myometrial closure performed, 5) suture material used for myometrial closure, 6) whether electrosurgery was used, and 7) length of time from myomectomy to conceiving.

Formalising these points using a standardised myomectomy operation note is a proposed start to collecting this data, reviewing how, if at all, we may be able to adjust our surgical techniques to improve future pregnancy outcomes and provide up-to-date counselling for women deciding on the mode of delivery and the length of time to wait before conceive following myomectomy. Makino et al. (2019) for example, published the Japanese uterine rupture survey reviewing cases of uterine rupture in Japan over the previous 5 years. They found that uterine rupture following previous myomectomy occurred at an earlier gestation (32 weeks) compared with those without previous uterine scar (39 weeks) and those with previous caesarean section (37 weeks). Neonatal prognosis in cases of uterine rupture in pregnancies following myomectomy was worse compared with those that occurred with previous caesarean section.

It should also be noted that in addition to useful prospective data collection for research, standardised operation notes have other non-academic benefits. For example, Oladipo et al. (2011) highlighted significantly improved documentation at a hospital level following the introduction of gynaecological surgical proformas, with improved legibility of notes and completeness of generic and procedure-specific items across all parameters measured. Standardised electronic operation notes also facilitate coding and can also enhance remuneration for hospitals (Theivendran et al., 2016). Moreover, operation notes form the only legal evidence of surgery being performed and substandard documentation results in limited data available for audit purposes. Inter- user heterogeneity of documentation may result in crucial missing information, posing a challenge to future clinicians to interpret. These documents also serve important medicolegal purposes.

Whilst other rejected items such as preoperative haemoglobin (anaemia known to be correlated with poorer surgical outcomes) (Fowler et al., 2015), use of anti-adhesives (with unknown potential impact on future fertility) and use of morcellation containment bags (to minimise the risk of inadvertently disseminating malignancy) are important for short- term and longer-term surgical outcomes, they are less useful in the context of a minimum dataset to correlate against obstetric outcomes.

### Strengths and Limitations

Our proposed standardised operation note incorporates advice from current literature and feedback from our questionnaire. The Royal College of Surgeons of England published official guidelines for operative note keeping improving the quality of procedure documentation (The Royal College of Surgeons of England, 2014). This guideline is considered the ‘gold standard’ for operative notes and can be tailored to any surgical specialty including gynaecology. Its use has been shown to improve the efficacy of surgical records for many years (Shayah et al., 2007). Mori et al. (1998) advise that section headers provide structure and order to the given area of documentation and should be included in a surgical proforma. Hoggett et al. (2017) outline five key headings needed to be included in any operation notes: Incision and approach, findings, procedure, closure, and postoperative instructions.

It was surprising that ethnicity was not selected by the majority of respondents as an important factor to be included in the data collection (only 46% favoured its inclusion) given its known prognostic importance in obstetric outcomes (Sheikh et al., 2022), not to mention inequity in type of surgical approach/techniques of fibroid surgical interventions (Orlando et al., 2022; Ptacek et al., 2021). We believe that this may reflect respondents focussing on items relevant to their surgical technique rather than appreciating that the ultimate goal of this operative data collection is to correlate with obstetric outcomes, thereby informing clinical decision making in an evidence- based manner.

Our survey was geographically limited to the Southeast of England, although National Health Service (NHS) data shows that over 40% of myomectomies in England are performed in London (Southeast UK) alone (Aref-Adib et al., 2023) so these findings should be applicable to the wider UK. We believe that these findings should also be generalisable internationally because the surgical approaches to myomectomy are well- established. Our overall response rate was only 44% from the total number of clinicians initially contacted by email, which limits the external validity of our findings (Yun and Trumbo, 2000). Due to the available resources, we did not optimise the potential response rate by sending reminders or using different methods of contacting clinicians e.g. text messages, QR codes, post, phone calls etc and this is a major limitation of or work. Despite this, our survey to our knowledge, is the largest reporting clinical opinions of both obstetricians and gynaecologists about factors to be considered for the design of a standardised operation note for myomectomy. Our response rate is also above the expected average response rate (35%) for physician specialists for web-based surveys (Cunningham et al., 2015).

### Next steps

Relevant stakeholders need to be involved in the implementation of a standardised operation note and this will be enhanced by utilising digital information technology. Education and training must be provided within units to ensure all users and readers of the document are aware of its existence and how to complete it. We propose utilising this document for a prospective database and associating this with pregnancy outcomes, allowing for large-scale data collection to gather evidence of complications of myomectomy in subsequent births. The value of such datasets have been highlighted in the Cumberlege (2020) report in the UK, resulting in the mandatory recording of mesh procedures in the British Society of Urogynaecology (BSUG) database. Even with gold standard databases such as the COMPARE-UF database (Stewart et al., 2018), the problem of missing data has been highlighted as something that needs further exploration to avoid compromising results (Munro, 2022). There are however databases that have been successful and have published meaningful outcome data sets correlating operative data with clinical outcomes e.g. the British Society for Gynaecological Endoscopy (BSGE) endometriosis database (Byrne et al., 2018). Hopefully with the use of this resource meaningful large-scale data can be collected following myomectomy.

## Conclusions

The need for future fertility is an important goal for many women who undergo uterine preserving interventions for leiomyomas such as myomectomy. To date, there no evidence- based guidelines addressing pregnancy outcomes following myomectomy (Amoah et al., 2022). Many women are advised to have an elective caesarean section based on little evidence (Odejinmi et al., 2020). The true risk of uterine rupture and other obstetric complications after myomectomy remains unknown. There is emerging evidence to suggest that prospective databases for informing women with fibroids of outcomes are beneficial to both patients and clinicians (Anchan et al., 2023).

Women deserve evidence-based guidance to facilitate an informed decision regarding mode and timing of delivery after myomectomy. A standardised operation note for open and laparoscopic myomectomy is in our opinion the first step towards to achieving this, by providing comprehensive, uniformly collected data to then correlate with subsequent fertility and obstetric outcomes. Practically, the use of standardised operation notes will also help improve accuracy of health records supporting the patient and operating surgeon should postoperative concerns arise, future pelvic surgery be indicated, or if medicolegal action is taken.

## Appendix 1: EXAMPLE STANDARDISED OPERATION NOTE FOR MYOMECTOMY

* Essential data to record according to survey respondents (>60% agreement rate), with a view to establishing a database to correlate previous myomectomy with pregnancy outcomes.
