# Supplementary material for: Current opinion on large-scale prospective myomectomy databases toward evidence-based preconception and antenatal counselling utilising a standardised myomectomy operation note
Source: Facts Views Vis Obgyn. 2024 Mar 28;16(1):59–65. doi: 10.52054/FVVO.16.4.006 (PMC11198879; doi:10.52054/FVVO.16.4.006)
Supplement: Supplementary file 1 [file FVVinObGyn-16-59-a001.pdf]

APPENDIX 1. EXAMPLE STANDARDISED OPERATION NOTE FOR MYOMECTOMY

*Essential data to record according to survey respondents (>60% agreement rate), with a view to establishing a database to correlate previous myomectomy with pregnancy outcomes.

Patient name: **Age*:**

Hospital number:

Primary operating surgeon: Surgical assistant/s:

Anaesthetist:

Procedure performed:

Indication for surgery*: Heavy menstrual bleeding [ ] Pressure effects [ ]

Fertility [ ] Other ________________

Patient demographics:

BMI* ______ Ethnicity __________________

Gravidity ______ Parity ______

Previous mode/s of delivery: Vaginal _________ Caesarean section _________

Previous fibroid surgery: None [ ] TCRF [ ]

Laparoscopic myomectomy [ ] Open myomectomy [ ]

Robotic myomectomy [ ]

Past Surgical History:

Past Medical History:

Findings

Vulva: Normal [ ] Abnormal [ ] _____________

Vagina: Normal [ ] Abnormal [ ] _____________

Cervix: Normal [ ] Abnormal [ ] _____________

Size of uterus: ____ / 40 weeks' gestation in size

Diagram

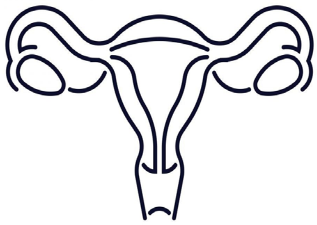

Entry & Approach

Technique used: Veress needle umbilical [ ] Veress needle palmer's point [ ]

Hassan technique [ ] Direct entry [ ]

Open: low transverse incision* [ ] Open: midline incision* [ ]

Port placement: Umbilical (5/8/10mm) [ ] Left lateral (5/8/10mm) [ ]

Right lateral (5/8/10mm) [ ] Suprapubic (5/8/10mm) [ ]

Palmer's point (5mm) [ ] Other:

Number of uterine incisions*: ___________

Electrosurgery used? Yes [ ] No [ ]

Morcellation used*? Yes: in bag [ ] Yes: no bag [ ] No [ ]

Location of uterine incisions*: Anterior [ ] Posterior [ ] Left lateral [ ]

Right lateral [ ] Fundal [ ] Cervical [ ]

Number of fibroids removed*: ________________

Location of largest fibroid removed* _____________

Size of largest fibroid removed* ____cm

Cavity breached*: Yes [ ] No [ ]

Posterior colpotomy performed*: Yes [ ] No [ ]

Suture material used for uterine closure*: _________________

Rectus sheath closure technique: _____________ Rectus sheath suture material _______

Skin closure technique: ____________ Skin closure material _______________

Drain inserted: No [ ] Yes [ ] type:_________________

Complications: No [ ] Yes [ ] type:_________________

Estimated Blood Loss*: ______mls

Additional procedures performed:

Right ovarian cystectomy [ ] Left ovarian cystectomy [ ] Adhesiolysis [ ]

Tubal-patency test [ ] Right salpingectomy [ ] Left salpingectomy [ ]

Right oophorectomy [ ] Left oophorectomy [ ]

Insertion of Copper intrauterine device [ ] Insertion of hormonal intrauterine system [ ]
